# Neuropeptide receptor genes *GHSR* and *NMUR1* are candidate epigenetic biomarkers and predictors for surgically treated patients with oropharyngeal cancer

**DOI:** 10.1038/s41598-020-57920-z

**Published:** 2020-01-23

**Authors:** Kiyoshi Misawa, Masato Mima, Yamada Satoshi, Yuki Misawa, Atsushi Imai, Daiki Mochizuki, Takuya Nakagawa, Tomoya Kurokawa, Miki Oguro, Ryuji Ishikawa, Yuki Yamaguchi, Shiori Endo, Hideya Kawasaki, Takeharu Kanazawa, Hiroyuki Mineta

**Affiliations:** 1 Department of Otolaryngology/Head and Neck Surgery, Hamamatsu University School of Medicine, Shizuoka, Japan; 2 Department of Otorhinolaryngology/Head and Neck Surgery, Graduate School of Medicine, Chiba University, Chiba, Japan; 3 Preeminent Medical Photonics Education & Research Center Institute for NanoSuit Research, Hamamatsu University School of Medicine, Hamamatsu, Japan; 4 Department of Otolaryngology, Tokyo Voice Center, International University of Health and Welfare, Tokyo, Japan

**Keywords:** Head and neck cancer, DNA methylation

## Abstract

Pathological staging and histological grading systems are useful, but imperfect, predictors of recurrence in head and neck squamous cell carcinoma (HNSCC). Aberrant promoter methylation is the main type of epigenetic modification that plays a role in the inactivation of tumor suppressor genes. To identify new potential prognostic markers, we investigated the promoter methylation status of five neuropeptide receptor genes. The methylation status of the target genes was compared with clinical characteristics in 278 cases; 72 hypopharyngeal cancers, 54 laryngeal cancers, 75 oropharyngeal cancers, and 77 oral cavity cancers were studied. We found that the *NTSR1, NTSR2, GHSR, MLNR*, and *NMUR1* promoters were methylated in 47.8%, 46.8%, 54.3%, 39.2%, and 43.5% of the samples, respectively. *GHSR* and *NMUR1* promoter methylation independently predicted recurrence in HNSCC. In patients with oropharyngeal cancer (n = 75), *GHSR* and *NMUR1* promoter methylation significantly correlates with survival in surgically treated patients. We classified our patients as having a low, intermediate, or high-risk of death based on three factors: HPV status, and *GHSR* and *NMUR1* promoter methylation. The disease-free survival (DFS) rates were 87.1%, 42.7%, and 17.0%, respectively. Combined data analysis of the methylation status of ten-eleven translocation (TET) family genes indicated a trend toward greater methylation indices as the number of TET methylation events increased. In the current study, we presented the relationship between the methylation status of the *GHSR* and *NMUR1* genes and recurrence in HNSCC, specifically in risk classification of oropharyngeal carcinomas cases with HPV status.

## Introduction

Head and neck squamous cell carcinoma (HNSCC) includes cancers of the pharynx, larynx, and oral cavity, and constitute approximately 4% of all cancers worldwide, with approximately 500,000 deaths annually^1^. Alcohol and tobacco consumption are the two most important risk factors for HNSCC, especially for cancers of the larynx, hypopharynx, and oral cavity^2^. Infection with various types of cancer-causing human papillomaviruses (HPV), primarily type 16, are risk factors for oropharyngeal cancers^3^. The 5-year overall survival rate of patients with HNSCC is approximately 40–50%^4^. Although HNSCC is a heterogeneous disease, the based on molecular studies have served to distinguish HPV-positive from HPV-negative HNSCC; however, validated molecular characterizations have not been established^5^.

G protein-coupled receptors (GPCRs) are the largest class of cell surface receptors involved in the development and progression of many cancers, including HNSCC^6^. GPCRs have highly druggable sites and comprise the largest class of pharmaceutical targets; at present, over 30% of FDA-approved drugs target GPCRs or their related pathways^7^. However, there are currently no anticancer drugs that specifically target GPCRs^8^. Frequent mutations of novel druggable oncogenes are not detected in HNSCC^9,10^. Epigenetic repression of GPCR genes correlates with worse prognosis and potential therapeutic targets^9,10^.

Whether patients with head and neck cancer who are regarded to be in the low-risk group could be saved the long-term complications of intensive, multimodal treatment without compromising their survival is now an extremely distinctive clinical question^11,12^. The initiation, progression, and resistance of cancer, traditionally considered a genetic disease, is now known to involve global epigenetic abnormalities, in addition to genetic alterations^13^. Epigenetic events are also potential drivers of acquired drug resistance in cancer^14^. Aberrant promoter methylation, an authentication of cancer cells, accounts for the inactivation of many tumor suppressor genes^15^. In a previous study, we found that ten-eleven translocation (*TET*) family genes of promoter region were aberrantly methylated in patients with HNSCC^16^. The five members of this family, TET1, TET2, and TET3, are enzymes that play a role of 5-methylcytosine oxidase and DNA demethylase^17^.

The principal aim of this study was to determine the methylation status of five GPCR-encoding genes in HNSCC and its association with survival and clinical parameters (e.g., tumor location and HPV status). All five genes, namely neurotensin receptor 1 (*NTSR1*), neurotensin receptor 2 (*NTSR2*), growth hormone secretagogue receptor (*GHSR*), motilin receptor (*MLNR*), and neuromedin U receptor 1 (*NMUR1*), encode neuropeptide receptors and belong to the Class Aβ subgroup clade 4. These five neuropeptide receptors have been implicated in the development of multiple types of cancer, but this study is the first to investigate their roles in the prognosis of HNSCC.

## Materials and Methods

### Tumor samples

Tissues were sampled (n = 278) from patients undergoing major surgical resection for HNSCC at the Department of Otolaryngology, Hamamatsu University School of Medicine (Hamamatsu, Shizuoka, Japan). All patients gave their written informed consent. Ethical clearance was received by the ethical committee of the Hamamatsu University School of Medicine (date of board approval: October 2, 2015, ethic code: 25–149), and informed consent was obtained from the participants. All methods were performed in accordance with the Declaration of Helsinki. The ratio of males to females was 233:45. The mean age was 65.4 years (age 32 to 92 years). Primary tumors were composed of 72 hypopharyngeal carcinomas, 54 laryngeal carcinomas, 75 oropharyngeal carcinomas, and 77 oral cavity carcinomas. Detailed clinical information was obtained from the patients’ medical records.

### DNA extraction and bisulfite modification

DNA was extracted from fresh specimens with a QIAamp DNA Mini Kit (Qiagen, Hilden, Germany) on the day of surgery. Purified genomic DNA was bisulfite-converted using the MethylEasy Xceed Rapid DNA Bisulfite Modification Kit (TaKaRa, Tokyo, Japan) following the manufacturer’s protocol.

### Quantitative methylation-specific PCR analysis (Q-MSP)

DNA methylation at CpG sites near promoter regions of the target genes was defined via quantitative methylation-specific PCR analysis **(**Q-MSP) using the Thermal Cycler Dice Real Time System TP800 (TaKaRa). The sequences of the primers used in this study are presented in Additional File 1: Table S1. Exon one and CpG sites within views of the promoter region relative to the transcription start site are presented in Additional File 2: Figure S1^18^. A standard curve for Q-MSP was constructed by plotting five serially diluted standard solutions of EpiScope Methylated HeLa gDNA (TaKaRa). The normalized methylation value (NMV) was defined as follows: NMV = (GPCRs gene-S/ GPCRs gene-FM)/(ACTB-S/ACTB-FM), where GPCRs gene-S and GPCRs gene-FM represent target gene methylation levels in the tumor sample and universal methylated DNA control, respectively. ACTB-S and ACTB-FM correspond to β-actin (ACTB) in the sample and the universally methylated DNA, respectively.

### Detection of high-risk HPV DNA by PCR

For high-risk HPV DNA detection, samples were assessed by PCR using specific primers for HPV types 16, 18, 31, 33, 35, 52, and 58. The prevalence of HPV DNA was analyzed with the PCR HPV Typing Set (TaKaRa). The PCR products were separated by electrophoresis on 9% polyacrylamide gels followed by staining with 0.5 g/mol ethidium bromide.

### Data mining in the Cancer Genome Atlas (TCGA)

The MethHC (http://methhc.mbc.nctu.edu.tw/php/index.php) was used to extract data from TCGA (available in April 2019)^19^. DNA methylation of GPCR genes was measured by Illumina Infinium Human Methylation 450 K BeadChip. The methylation score for each CpG site is represented as β values and ranges from 0 to 1, corresponding to unmethylated and completely methylated DNA, respectively.

### Data analysis and statistics

The receiver-operator characteristic (ROC) curve analysis was used to evaluate the NMVs for 36 matched paired HNSCC and normal mucosal samples and the Stata/SE 13.0 system (Stata Corporation, TX, USA). In an area under the ROC curve, the true positive rate (Sensitivity) is plotted as a function of the false positive rate (1-Specificity) for different cutoff points, and the NMV thresholds were estimated for each target gene. Cutoff values showing the greatest accuracy were determined based on sensitivity/specificity, as indicated in Additional File 3: Table S2. We used the cutoff values to determine the methylation frequencies of the target genes. Calculation of the methylation index (MI) was defined as the ratio between the number of methylated genes and the number of tested genes in all the samples^16^.

Associations between the clinical variables were analyzed using the Student’s t-test. Disease-free survival (DFS) probabilities were estimated using the Kaplan-Meier method, and the log-rank test was applied to assess the significant differences among actuarial survival curves. Cox’s proportional hazards regression analysis that included age (≥65 vs. <65 years), sex, alcohol intake, smoking status, and tumor stage (I–II vs. III–IV), and the methylation status was used to identify the multivariate predictive value of the prognostic factors^20^. P < 0.05 was considered statistically significant.

### Ethics approval and consent to participate

The research methodology employed in this study was approved by the Institutional Review Board of the Hamamatsu University School of Medicine. All study subjects provided written informed consent.

## Results

### Analysis of the methylation status of HNSCC tissue samples

**Figure 1:**
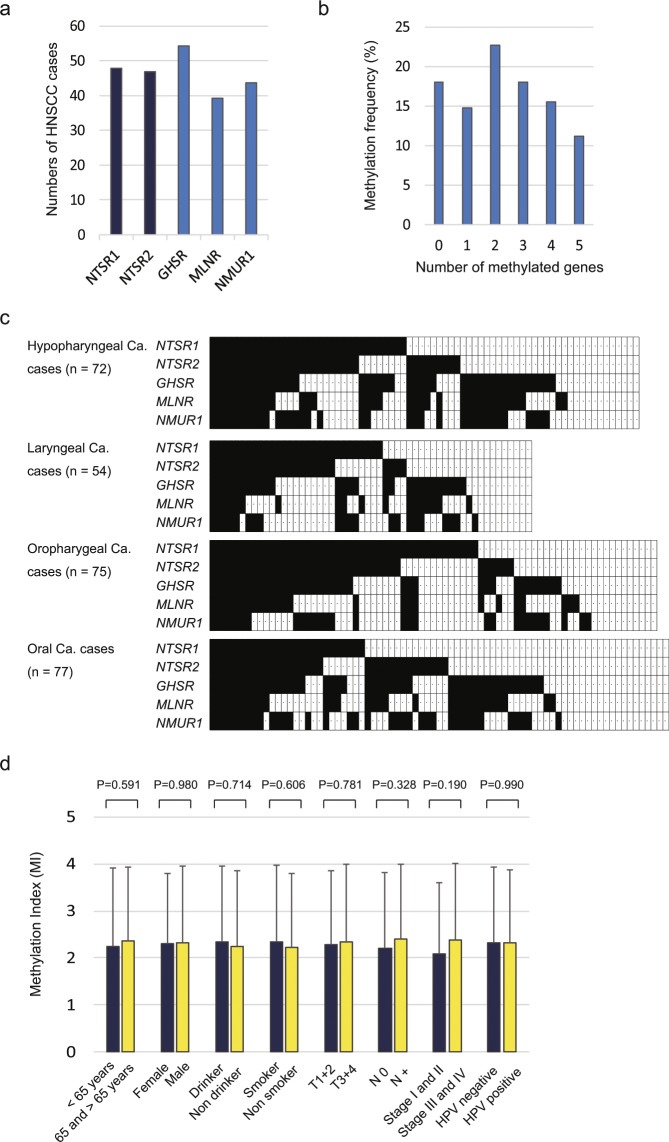
Methylation of the neuropeptide receptor gene promoters in 278 HNSCC samples. (**a**) Bar graph showing the methylation frequencies of the five genes. (**b**) Bar graph showing the percentage of tumors that express zero to five methylated target genes. (**c**) Comparison of the methylation status of the promoters of the five genes in patients with hypopharyngeal, laryngeal, oropharyngeal, or oral cancer. Filled boxes indicate the presence of methylation, and open boxes indicate the absence of methylation (**d**) Bar graph showing the methylation indices (MIs) according to selected clinical parameters. The mean MI for each parameter was determined by the Student’s t-test.

Q-MSP analysis was used to determine the methylation status of five genes encoding GPCR neuropeptide receptors in 278 primary HNSCC samples and it was a valuable test. The methylation frequencies are as follows: *NTSR1* (47.8%), *NTSR2* (46.8%), *GHSR* (54.3%), *MLNR* (39.2%), and *NMUR1* (43.5%) (Fig. 1a). The average number of methylated genes per sample was 2.32 ± 1.61(range: 0–5) (Fig. 1b). Primary tumors were located in the hypopharynx (n = 72), larynx (n = 54), oropharynx (n = 75), or oral cavity (n = 77) (Fig. 1c). *NTSR1*, *NTSR2*, *GHSR*, *MLNR*, and *NMUR1* promoter hypermethylation presented discriminative ROC curve profiles, which clearly differentiate cancer tissues from normal tissues [Area Under Curve (AUC) = 0.6220, 0.5736, 0.8103, 0.6049, and 0.5631, respectively] (Additional File 4: Fig. S2). A specimen was classified as methylated when its NMV exceeded 0.045, 0.009, 0.563, 0.700, and 0.735 for *NTSR1*, *NTSR2*, *GHSR*, *MLNR*, and *NMUR1*, respectively (Additional File 3: Table S2). *NTSR1*, *NTSR2*, *GHSR*, *MLNR*, and *NMUR1* methylation levels in primary HNSCCs were significantly higher than those in matched paired normal mucosal tissues (Additional File 5: Fig. S3).

### Clinicopathological characteristics of primary HNSCC samples

The MI was determined as the number of methylated genes to the number of tested genes in each sample. No significant differences in MI were observed regarding the age at disease onset, sex, alcohol consumption, smoking status, tumor size, lymph node status, clinical stage, or HPV status (Fig. 1d). Associations between the methylation status of the target genes and the clinical characteristics of the patients are shown in Additional File 6: Table S3. Except for HPV status, there is no significant association between neuropeptide receptor gene promoter hypermethylation and clinicopathological parameters. Methylation of the *NTSR1* and *GHSR* promoters are significantly correlated with HPV status (P = 0.004 and P = 0.038, respectively) (Additional File 6: Table S3). Correlations between HPV status and primary sites are shown in the Additional File 7: Table S4.

### Comparison of methylation frequencies for five neuropeptide receptor genes and *TET* family genes

**Figure 2:**
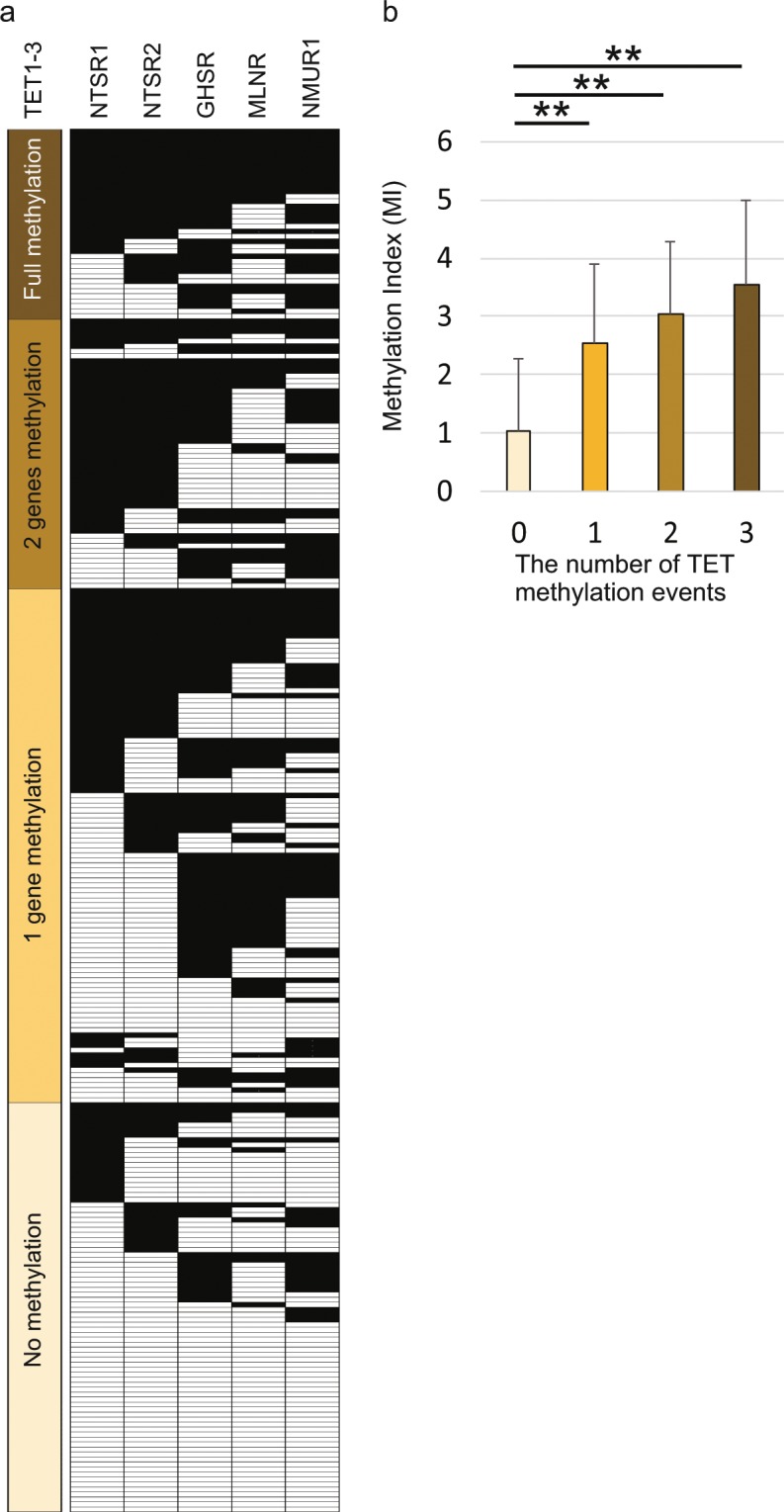
Correlation between promoter methylation levels of the five neuropeptide receptor genes and *TET* family genes in cancer tissues. (**a**) Distribution of promoter methylation in *TET* family genes and the five neuropeptide receptor genes. Filled boxes indicate the presence of methylation, and open boxes indicate the absence of methylation. (**b**) Combined analysis of the MIs and methylation status of *TET* family genes. The number of methylation events is indicated for hypermethylated *TET* family genes. The mean MIs for the different groups were compared using the Student’s t-test. **P < 0.001. The data are shown as mean ± SD.

Mean differences in the methylation index of the five GPCR neuropeptide receptors determined based on *TET* gene methylation events are illustrated in Fig. 2a. The MI was significantly higher in patients with full *TET* genes methylation events (3.55 ± 1.43), two *TET* genes methylation events (3.03 ± 1.26), and one *TET* gene methylation events (2.53 ± 1.38) than in patients with no *TET* gene methylation events (1.02 ± 1.26, P < 0.001 for all comparisons) (Fig. 2b).

### Kaplan-meier estimate

**Figure 3:**
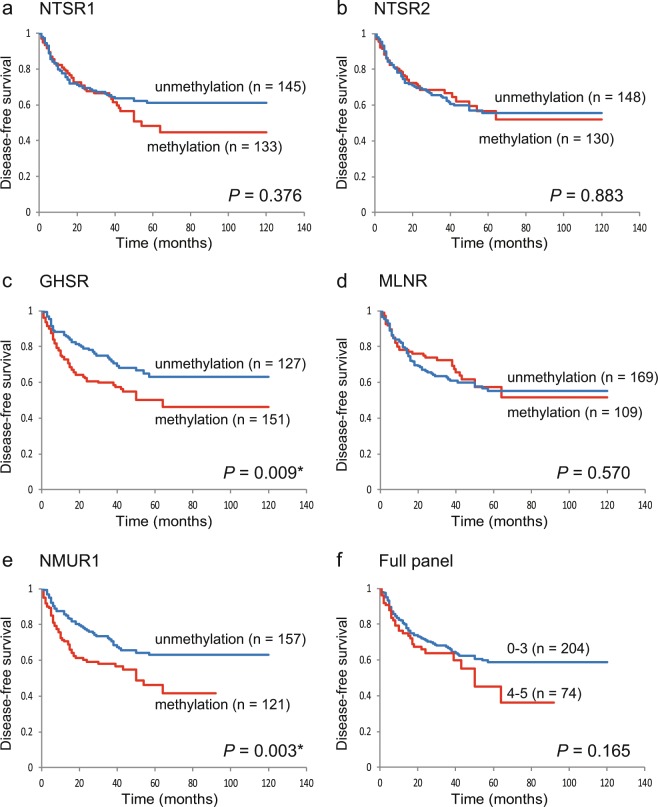
Kaplan-Meier survival curves for the 278 patients with HNSCC according to the methylation status of the five target genes. DFS for (**a**) *NTSR1*, (**b**) *NTSR2*, (**c**) *GHSR*, (**d**) *MLNR*, and (**e**) *NMUR1* in the case of methylated (red lines) and unmethylated (blue lines) genes. (**f**) Combined analysis of the five genes. Blueline: patients with 0–3 methylated genes; red line: patients with 4–5 methylated genes. A probability of <0.05 (*P < 0.05) was considered a statistically significant difference.

The Kaplan-Meier analysis of the DFS is shown in Fig. 3. DFS did not differ between in patients with methylated and unmethylated genes (Fig. 3a,b,d,f), with a few notable exceptions: DFS was significantly shorter when the *GHSR* (log-rank test, P = 0.009) and *NMUR1* (log-rank test, P = 0.003) promoters were methylated (Fig. 3c,e). Among 135 cases with T1 and T2 tumor sizes, the DFS rates in those with *GHSR* and *NMUR1* methylated genes were compared to the unmethylated group (log-rank test, P = 0.418 and P = 0.031, respectively) (Additional File 8: Fig. S4a,b). It was found that patients with T1 and T2 tumor sizes and methylated *NMUR1* promoters had shorter DFS. Additional analysis that included only patients with oropharyngeal cancer (n = 75) revealed shorter DFS for methylated vs. unmethylated *GHSR* and *NMUR1* (log-rank test, P = 0.004 and P = 0.008, respectively), but no differences for the other three genes (Additional File 8: Fig. S4c,d). Furthermore, in HPV-related oropharyngeal cancer (n = 37), *GHSR* and *NMUR1* hypermethylation was significantly associated with shorter DFS (log-rank test, P = 0.003 and P = 0.026, respectively) (Additional File 8: Fig. S4e,f).

### Stratification analysis

**Figure 4:**
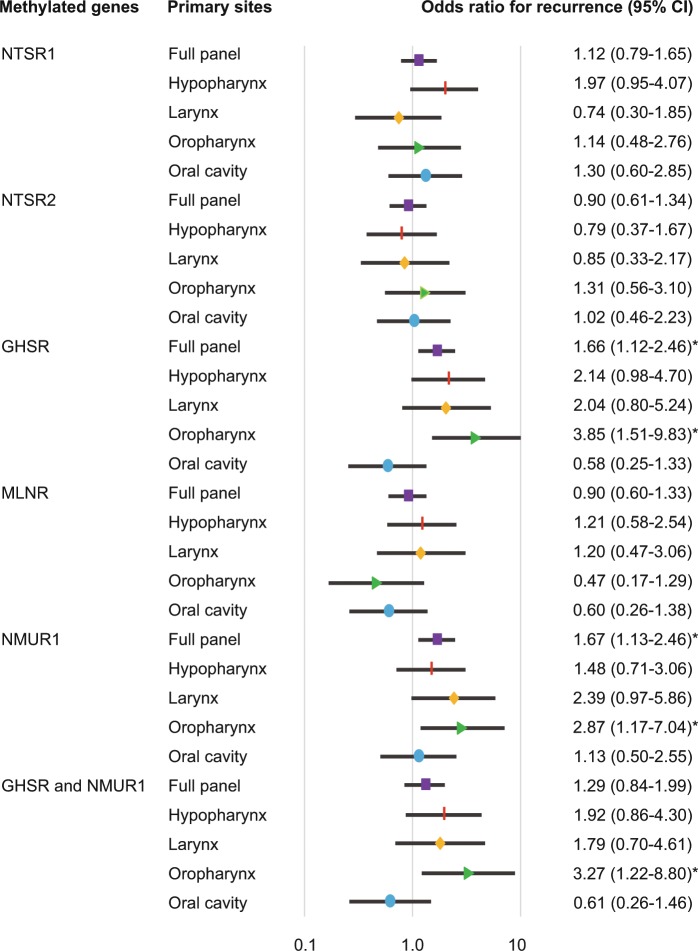
Risk of recurrence based on gene methylation in tumors with different origins. Odds ratios for recurrence were determined using a Cox proportional hazards model adjusted for age (≥65 vs. <65 years), sex, smoking status, alcohol intake, and tumor stage (I–II vs. III–IV). CI: confidence interval.

The relation between methylation and risk of recurrence was analyzed through multivariate analysis using a Cox proportional hazards regression model adjusted for age, sex, smoking status, alcohol consumption, and clinical stage. In patients with *GHSR* promoter methylation, the adjusted odds ratio (OR) for recurrence was 1.656 [95% confidence interval (CI) 1.116–2.459, P = 0.012]. *NMUR1* promoter methylation had a significant association with the OR for recurrence (OR = 1.670, 95% CI 1.133–2.458, P = 0.009) (Fig. 4). The OR for recurrence according to original tumor sites, was also determined. Methylation of the *GHSR* and *NMUR1* promoters correlated positively with recurrence in oropharyngeal cancer patients, both individually (OR, 3.853; 95% CI, 1.510–9.832; P = 0.005 and OR, 2.872; 95% CI, 1.172–7.037; P = 0.036, respectively) and together (OR, 3.272; 95% CI, 1.216–8.801; P = 0.019) (Fig. 4).

**Figure 5:**
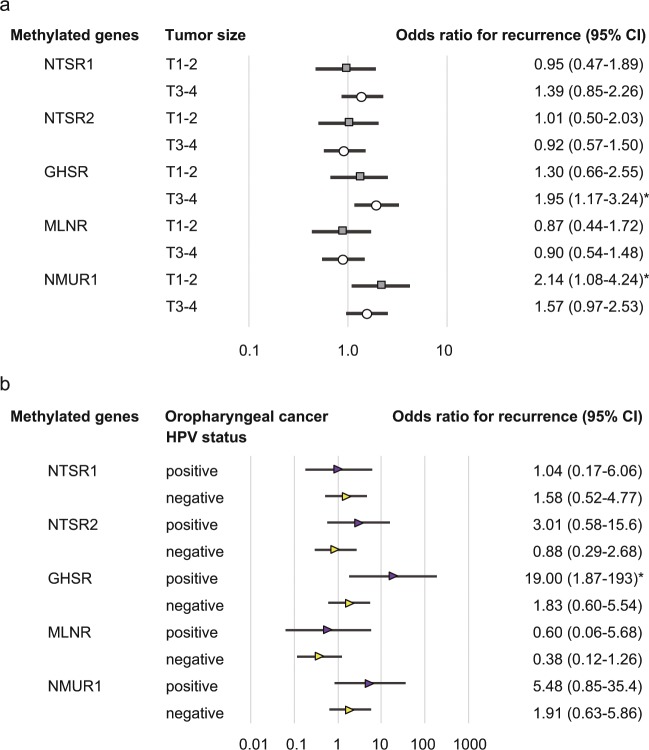
Odds ratios for recurrence based on the Cox proportional hazards model. Multivariate Cox regression analyses were performed to assess the correlations between (**a**) recurrence and patients with T1–2 (n = 135) and T3–4 tumor sizes (n = 143) and between (**b**) recurrence and patients with HPV-positive (n = 37) and HPV-negative oropharyngeal cancer (n = 38).

In patients with T1 and T2 tumor sizes, *NMUR1* promoter methylation has revealed a significant association with the OR for recurrence (OR = 2.14, 95% CI: 1.08–4.24, P = 0.028). For patients with T3 and T4 tumor sizes with a methylated *GHSR* promoter, the OR was 1.95 (95% CI: 1.17–3.24; P = 0.010) (Fig. 5a). Notably, the OR was significantly higher in HPV-positive oropharyngeal cancer patients (n = 37) in whom the *GHSR* promoter was methylated (OR, 19.00; 95% CI, 1.87–193.01; P = 0.013) (Fig. 5b).

### Multivariate analysis including HPV status and methylation status in oropharyngeal cancer patients

**Figure 6:**
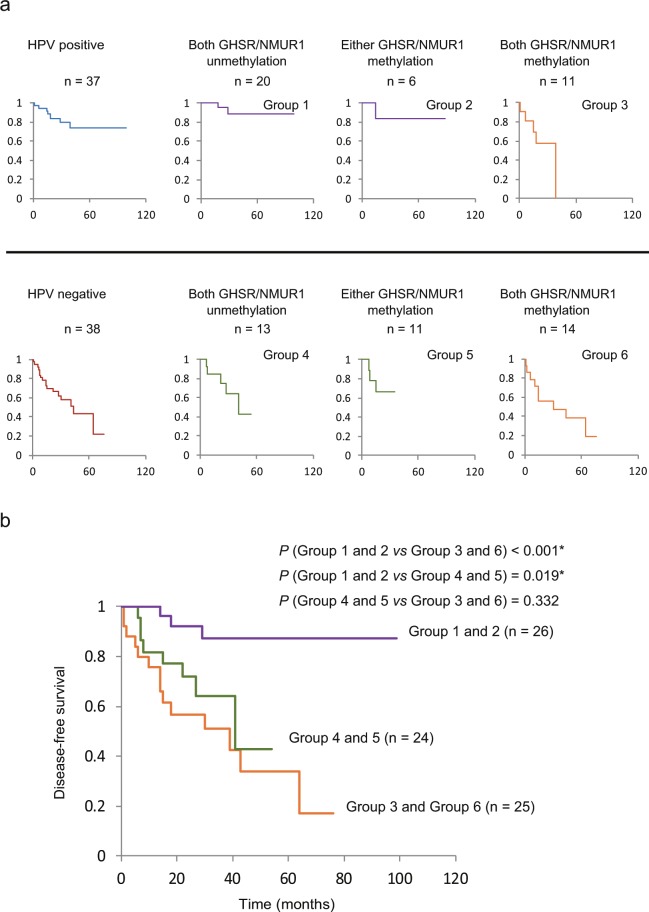
Classification of study patients with oropharyngeal carcinoma into the risk of recurrence categories and Kaplan-Meier estimates of DFS according to the categories. (**a**) Kaplan-Meier estimates of DFS among oropharyngeal cancer patients to classify patients into categories of low-, intermediate-, or high-risk of recurrence, according to *GHSR* methylation, *NMUR1* methylation, and HPV status. (**b**) Patients with oropharyngeal carcinoma were classified into three categories with respect to the risk of recurrence. Group 1 and 2: low-risk group; Group 4 and 5: intermediate-risk group; Group 3 and 6: high-risk group.

For correlation analysis of the association between tumor HPV status, *GHSR* methylation status, and *NMUR1* methylation status with survival, we combined data for all patients with oropharyngeal carcinoma. The DFS was correlated to better outcomes for patients with HPV-positive cancers than those with HPV-negative cancers (log-rank test, P = 0.017) (Fig. 6a). The study patients were classified into three categories with respect to the risk of recurrence: low-risk (Group 1 and 2), HPV-positive with both *GHSR* and *NMUR1* unmethylated, or either *GHSR* or *NMUR1* methylated; intermediate-risk (Group 4 and 5), HPV-negative with both *GHSR* and *NMUR1* unmethylated, or either *GHSR* or *NMUR1* methylated; and high-risk (Group 3 and 6), any HPV status with both *GHSR* and *NMUR1* methylated. HPV-positive cancer patients were regarded to be at low-risk, with the exception of patients with both *GHSR* and *NMUR1* methylated (Fig. 6a). As shown in Fig. 6b, DFS rates in the patients were 87.1% (95% CI, 73.4–100%), 42.7% (95% CI, 5.4–80.1%), and 17.0% (95% CI, 0–43.4%), for the low-risk, intermediate-risk, and high-risk groups, respectively. DFS was statistically significant different across risk groups (P < 0.001 for low- vs. high-risk and P = 0.019 for low- vs. intermediate-risk) (Fig. 6b).

### External validation of our results using methylation data from the TCGA database

The methylation status of GPCR neuropeptide receptor gene promoters was estimated in an additional 516 HNSCC samples and 50 normal samples from the database. The average β values of promoter methylation for the five genes were significantly higher in the HNSCC samples than in the normal samples (P < 0.001) (Additional File 9: Fig. S5a). The validation of TCGA data was used to assess the methylation status of the five genes in tumors from the hypopharynx, larynx, oropharynx, or oral cavity (Additional File 9: Fig. S5b; Additional File 9: Fig. S5c). The mRNA expression status of the five neuropeptide receptor genes in HNSCC and normal samples were obtained from the TCGA database (Additional File 10: Fig. S6).

## Discussion

GPCRs belong to a superfamily of cell surface signaling proteins that have an important role in many physiological functions and multiple diseases, including the development of malignant neoplastic disease^21^. We found that aberrant methylation of the *GHSR* and *NMUR1* promoters correlates with survival and recurrence in patients with HNSCC. In addition, the site-specific analysis revealed that abnormal CpG island hypermethylation in the *GHSR* and *NMUR1* promoters was independently associated with aggressive clinical behavior in oropharyngeal cancer. It is worth noting that the *GHSR* and *NMUR1* methylation status is a strong predictor of poorer survival among patients with HPV-positive oropharyngeal cancer.

The GPCR family members include the neurotensin receptors, of which there are two subtypes, NTSR1 and NTSR2, which are associated with carcinogenesis, cancer progression, and prognosis^22–24^. Neurotensin is a 13-amino acid neuropeptide that is localized principally in the central nervous system^25^. The actions of neurotensins are mediated through a high-affinity receptor (NTSR1) and a low-affinity receptor (NTSR2)^26^. In neuroendocrine tumors, a lack of *NTSR1* promoter methylation with overexpression and dense of *NTSR2* promoter methylation are observed^24^. On the contrary, *NTSR1* methylation is related to lateral and noninvasive tumor growth of colorectal tumors^27^.

GHSR is also known as the ghrelin receptor, and the hormone ghrelin is its endogenous ligand. Strikingly, our study identified a single locus within the promoter region of the *GHSR* gene that is hypermethylated in 54.3% (151 of 278) of HNSCC, independently of patient age or tumor stage. *GHSR* promoter methylation was the most accurately detected (with AUROC of 0.81 obtained from the ROC) in tumor samples and matched paired normal mucosal samples. Loss of *GHSR* expression correlates with hypermethylation of *GHSR* in breast, cervical, prostate, pancreatic, colorectal, and pharyngeal cancers, and glioblastoma^28–31^. *GHSR* methylation in cervical tissue and scrapes is associated with 3q gain for the detection of HPV-induced cervical precancer^29^. These findings indicate that *GHSR* methylation may represent a potential pancancer marker for the detection of multiple tumor types including HNSCC (Additional File 11: Table S5).

Motilin is a gastrointestinal hormone released from the duodenum^32^. The MLNR shares significant amino acid sequence identity with GHSR^33^. Recently, it was reported that variants in the *MLNR* gene (rs9568169) are specific genetic risk factors for bile duct cancer^34^. Neuromedin U and its structurally related peptide, neuromedin S, are reported to regulate multiple physiological processes, and their functions are mediated by two receptors, NMUR1 and NMUR2. In a DNA methylation analysis to elucidate the potential molecular mechanisms underlying osteosarcoma, *neuromedin U* and *NMUR1* methylation exhibited the highest degrees selected from the protein-protein interaction network^35^. The *NMUR2* promoter region is C+ G-rich; however, the level of condensation of CpG sequences in this region is too low to design primers for a methylation assay^36^.

CpG hypermethylation is a major epigenetic DNA modification that tumor suppresses gene expression in cancer tumorigenesis and progression^37^. Analysis of genomic structure showed a CpG island in a region containing translational start sites that extends to the first exon of *NTSR1*, *NTSR2*, *GHSR*, *MLNR*, and *NMUR1*. Proper DNA methylation depends on the underlying mechanisms that regulate the writing, reading, and erasing of methylation marks^38^. Interestingly, the activity of TET enzymes is involved in removing epigenetic methylation marks^39^. TET proteins prevent unwanted DNA methyltransferase activity by binding to CpG-rich regions^40^. Recently, we reported that TET inactivation through promoter methylation occurs in HNSCC and promotes the inactivation of tumor suppressor genes^16^.

HPV-related oropharyngeal carcinomas belong to an independent tumor type with regard to cellular, biological, and clinical features^41^. HPV status, smoking status, tumor stage, and lymph node status are important factors that can be used to classify patients with low-, intermediate-, and high-risk of death^11,42–44^. However, the optimal classification for patients with low-, intermediate-, and high-risk disease remains to be determined. Our findings suggest that it is important to integrate HPV status and methylation status as determinants of recurrence risk for patients with oropharyngeal carcinoma.

## Conclusion

We have shown that high-throughput methylation profiles can be correlated with recurrence and survival in HNSCC. Our results indicate that the methylation status of the *GHSR* and *NMUR1* genes is an independent prognostic indicator for patients with oropharyngeal cancers. Furthermore, the function of *TET* genes as a methylation eraser should be considered in future studies of HNSCC carcinogenesis and its potential biomarkers and therapeutic targets. Our findings support the use of methylation markers in patient selection for adjuvant therapy following primary treatment with surgery and oropharyngeal cancer surveillance programs. Since our study is preliminary, it needs to be validated in larger cohorts of patients with more homogeneous oropharyngeal cancer patients.

## Supplementary information

Supplementary information
