# Supplementary material for: Neuropeptide receptor genes GHSR and NMUR1 are candidate epigenetic biomarkers and predictors for surgically treated patients with oropharyngeal cancer
Source: Sci Rep. 2020 Jan 23;10:1007. doi: 10.1038/s41598-020-57920-z (PMC6978330; doi:10.1038/s41598-020-57920-z)
Supplement: Supplementary file 1 — Supplementary information [file 41598_2020_57920_MOESM1_ESM.pdf]

Neuropeptide receptor genes *GHSR* and *NMUR1* are candidate epigenetic biomarkers and predictors for surgically treated patients with oropharyngeal cancer.

Kiyoshi Misawa, Masato Mima, Yamada Satoshi, Yuki Misawa,Atsushi Imai,

Daiki Mochizuki, Takuya Nakagawa, Tomoya Kurokawa, Miki Oguro, Ryuji Ishikawa,

Yuki Yamaguchi, Shiori Endo, Hideya Kawasaki, Takeharu Kanazawa, Hiroyuki Mineta

SUPPLEMENTARY INFORMATION

Additional file 1: Table S1. Q-MSP primer list

Gene	Forward primer 5'-3'	Reverse primer 5'-3'	Base pairs
NTSR1	TGCGTTTTAATAGTTTCGCGT	CGTTACCGAAACCCGAAACCAACA	111
NTSR2	GTCGCGGGAGCGGGATGGAAATTA	AACGAATATCCACGCCCAACCGA	99
GHSR	TAGTATGTGGAACGCGACGT	AACTCGTCGCCCAACGAATC	105
MLNR	ATCGAGAGGGATTACGCGTT	AAACCGTCGCTACCGTTCCA	97
NMUR1	GCGCGGGTTGGGGTCGTTGT	CGCACCTACCATACGACCCG	114
TET1	ATCGGCGCGAGTTGGAAAGTT	GACCCCAACTCACCGCTAACCG	103
TET2	CGCGGGTAACGGGATTTAAAG	GTACCCTCGCTCTAACCCCCG	123
TET3	CGAGGGGGTGGAGATGGTCGAAAGAAAC	CGTACGACGATTAATACAACT	108
ACTB	TGGTGATGGAGGAGGTTTAGAAGT	AACCAATAAAACCTACTCCTCCCTTAA	133

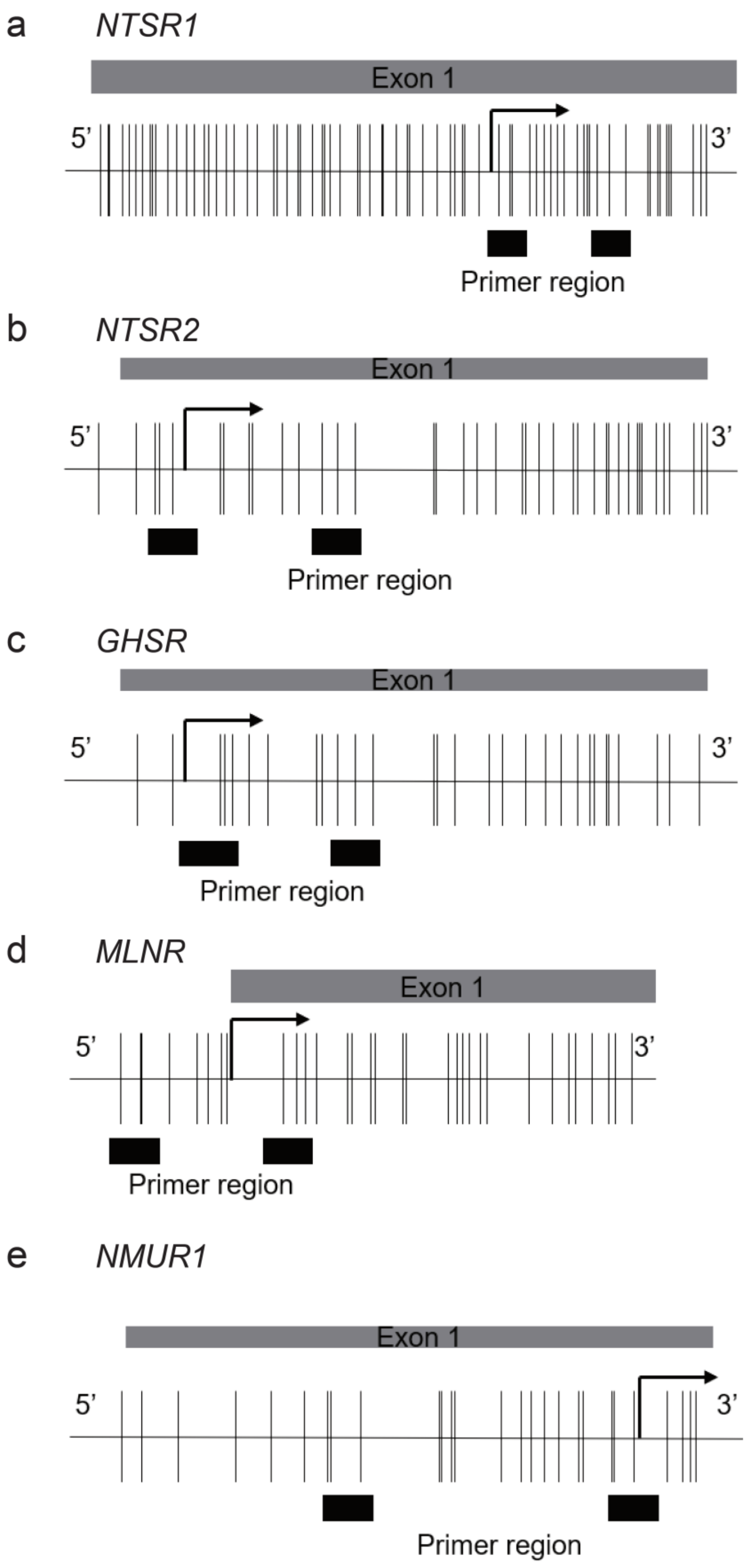
a
NTSR1
Exon 1
5'
3'
Primer region
b
NTSR2
Exon 1
5'
3'
Primer region
c
GHSR
Exon 1
5'
3'
Primer region
d
MLNR
Exon 1
5'
3'
Primer region
e
NMUR1
Exon 1
5'
3'
Primer region

Additional file 2: Figure S1. Schematic representation of *NTSR1*, *NTSR2*, *GHSR*, *MLNR,* and *NMUR1* genes

CpG sites are within the expanded views of the promoter region. Vertical lines, individual CpG sites; black box, the relative location of the primers used for Q-MSP; curved arrow, translation start site (ATG).

Additional file 3: Table S2. Results of the ROC curve analysis, the sensitivity, specificity, and cutoff value.

Genes	ROC Area	Sensitivity (%)	Specificity (%)	Cutoff value
NTSR1	0.622	70.31	50.00	0.045
NTSR2	0.5736	75.00	36.00	0.009
GHSR	0.8103	58.33	93.75	0.563
MLNR	0.6049	25.00	97.22	0.700
NMUR1	0.5631	26.53	97.96	0.735

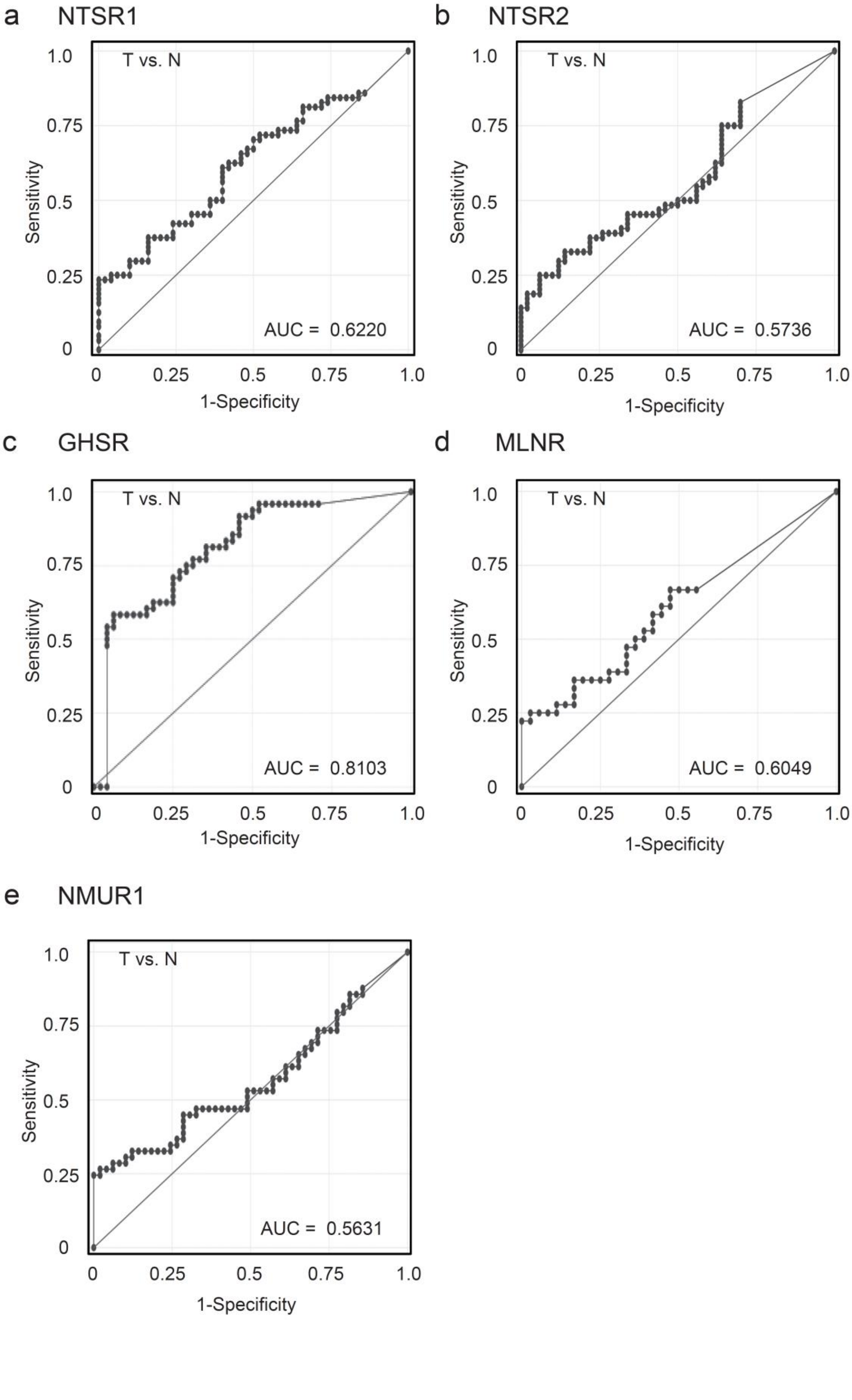
a
NTSR1
T vs. N
AUC = 0.6220
b
NTSR2
T vs. N
AUC = 0.5736
c
GHSR
T vs. N
AUC = 0.8103
d
MLNR
T vs. N
AUC = 0.6049
e
NMUR1
T vs. N
AUC = 0.5631
Sensitivity
1-Specificity
0
0.25
0.5
0.75
1.0

Additional file 4: Figure S2. Receiver operating characteristic (ROC) curves for the methylation markers in cancer tissue versus adjacent normal mucosal tissue

Based on the ROC curve analysis, AUCs are 0.6220 for *NTSR1* (a), 0.5736 for *NTSR2* (b), 0.8103 for *GHSR* (c), 0.6049 for *MLNR* (d), and 0.5631 for *NMUR1* (e).

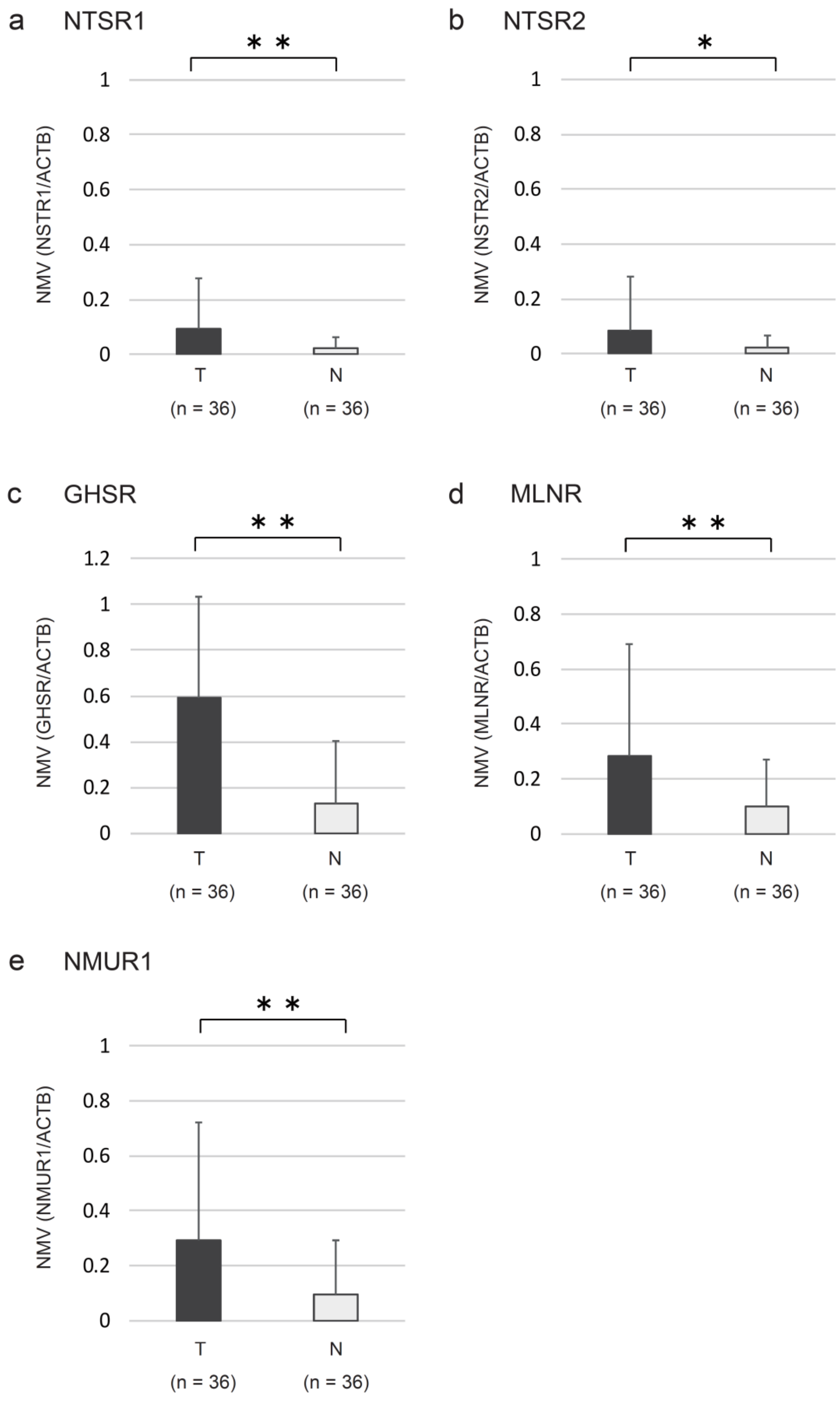

a
NTSR1
* *
NMV (NSTR1/ACTB)
1
0.8
0.6
0.4
0.2
0
T
(n = 36)
N
(n = 36)
b
NTSR2
*
NMV (NSTR2/ACTB)
1
0.8
0.6
0.4
0.2
0
T
(n = 36)
N
(n = 36)
c
GHSR
* *
NMV (GHSR/ACTB)
1.2
1
0.8
0.6
0.4
0.2
0
T
(n = 36)
N
(n = 36)
d
MLNR
* *
NMV (MLNR/ACTB)
1
0.8
0.6
0.4
0.2
0
T
(n = 36)
N
(n = 36)
e
NMUR1
* *
NMV (NMUR1/ACTB)
1
0.8
0.6
0.4
0.2
0
T
(n = 36)
N
(n = 36)

Additional file 5: Figure S3. Hypermethylation patterns in 36 matched pairs of head and neck tumors and adjacent normal mucosal tissues

The NMVs for the *NTSR1* (a), *NTSR2* (b), *GHSR* (c), *MLNR* (d), and *NMUR1* (e) promoters were significantly higher in head and neck tumor tissues (T) than in paired adjacent normal mucosal tissue (N) with P = 0.009, 0.032, < 0.001, 0.012, and 0.007, respectively. The differences were significant, as determined by the Student's t-test. *P < 0.05. **P < 0.01.

Additional file 6: Table S3. Results of the ROC curve analysis, the sensitivity, specificity, and cutoff value.

	Gene	NTSR1			NTSR2			GHSR			MLNR			NMUR1		
	Methylation status	Yes	No	P †	Yes	No	P †	Yes	No	P †	Yes	No	P †	Yes	No	P †
Characteristics	Overall (%)	133 (47.8%)	145 (52.2%)		130 (46.7%)	148 (53.3%)		151 (54.3%)	127 (45.7%)		109 (39.2%)	169 (60.8%)		121 (43.5%)	157 (56.5%)	
Age	< 65	55	59		53	61		59	55		46	68		44	70	
	> 65	78	86	1	77	87	1	92	72	1	63	101	1	77	87	0.178
Gender	Female	21	24		21	24		22	23		17	28		23	22	
	Male	112	121	0.872	109	124	1	129	104	0.514	92	141	0.869	98	135	0.325
Smoking status	smoker	102	110		103	109		117	95		82	130		93	119	
	non smoker	31	35	0.889	27	39	0.323	34	32	1	27	39	1	28	38	0.887
Alcohol exposure	drinker	101	110		102	109		116	95		85	126		89	122	
	non drinker	32	35	1	28	39	0.4	35	32	1	24	43	0.567	32	35	1
Tumor size	T1-2	64	71		64	71		74	61		53	82		54	81	
	T3-4	69	74	0.905	66	77	1	77	66	0.904	56	87	1	67	76	0.277
Lympho-node status	N0	50	60		46	64		58	52		43	67		45	65	
	N+	83	85	0.541	84	84	0.219	93	75	1	66	102	1	76	92	0.537
Stage	I, II	28	34		23	39		33	29		23	39		22	40	
	III, IV	105	111	0.667	107	109	0.112	118	98	1	86	130	0.769	99	117	0.191
HPV status	positive	32	15		26	21		19	28		14	33		18	29	
	negative	101	130	0.004*	104	127	0.204	132	99	0.038*	95	136	0.19	103	128	0.519

† Fisher's exact test * $P<0.05$

Additional file 7: Table S4. HPV status and primary sites.

Primary sites	HPV positive	HPV negative	Total
Hypopharyngeal Cancers	5 (6.9%)	67 (93.1%)	72 (100%)
Laryngeal Cancers	2 (3.7%)	52(96.3%)	54 (100%)
Oropharygeal Cancers	37 (49.3%)	38(50.7%)	75 (100%)
Oral Cancers	3 (3.9%)	74(96.1%)	77 (100%)
Total	47 (16.9%)	231(83.1%)	278 (100%)

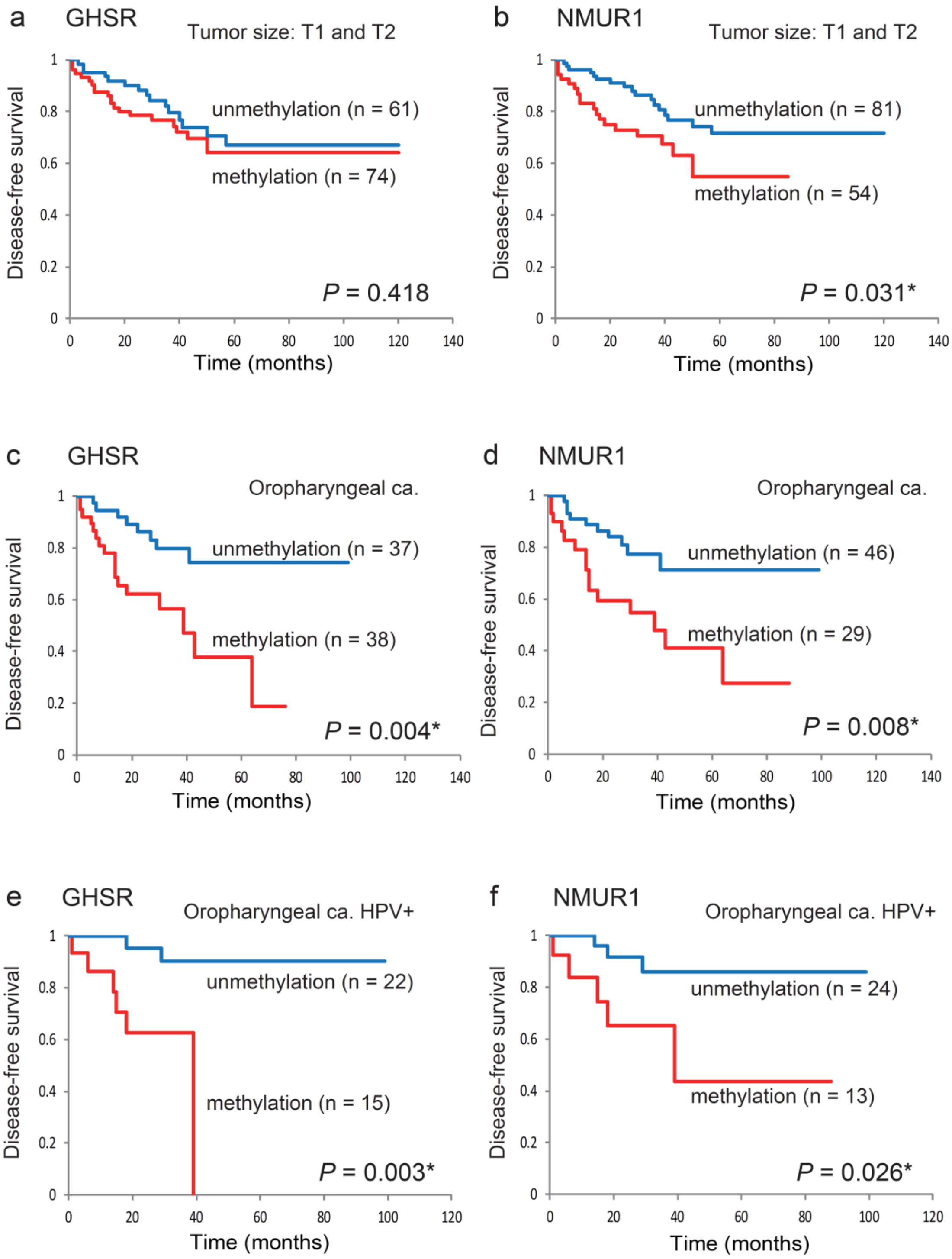
a
GHSR
Tumor size: T1 and T2
unmethylation (n = 61)
methylation (n = 74)
P = 0.418
b
NMUR1
Tumor size: T1 and T2
unmethylation (n = 81)
methylation (n = 54)
P = 0.031*
c
GHSR
Oropharyngeal ca.
unmethylation (n = 37)
methylation (n = 38)
P = 0.004*
d
NMUR1
Oropharyngeal ca.
unmethylation (n = 46)
methylation (n = 29)
P = 0.008*
e
GHSR
Oropharyngeal ca. HPV+
unmethylation (n = 22)
methylation (n = 15)
P = 0.003*
f
NMUR1
Oropharyngeal ca. HPV+
unmethylation (n = 24)
methylation (n = 13)
P = 0.026*
Disease-free survival
Time (months)

Additional file 8: Figure S4. Kaplan-Meier survival curves

Kaplan-Meier survival curves for *GHSR* and *NMUR1* in (a and b) patients with T1–2 tumor sizes (n = 135), (c and d) patients with oropharyngeal cancer (n = 75), and (E and F) patients with HPV-positive oropharyngeal cancer (n = 37). The log-rank test was used to compare the survival times between patients with methylated (red lines) and unmethylated (blue lines) genes.

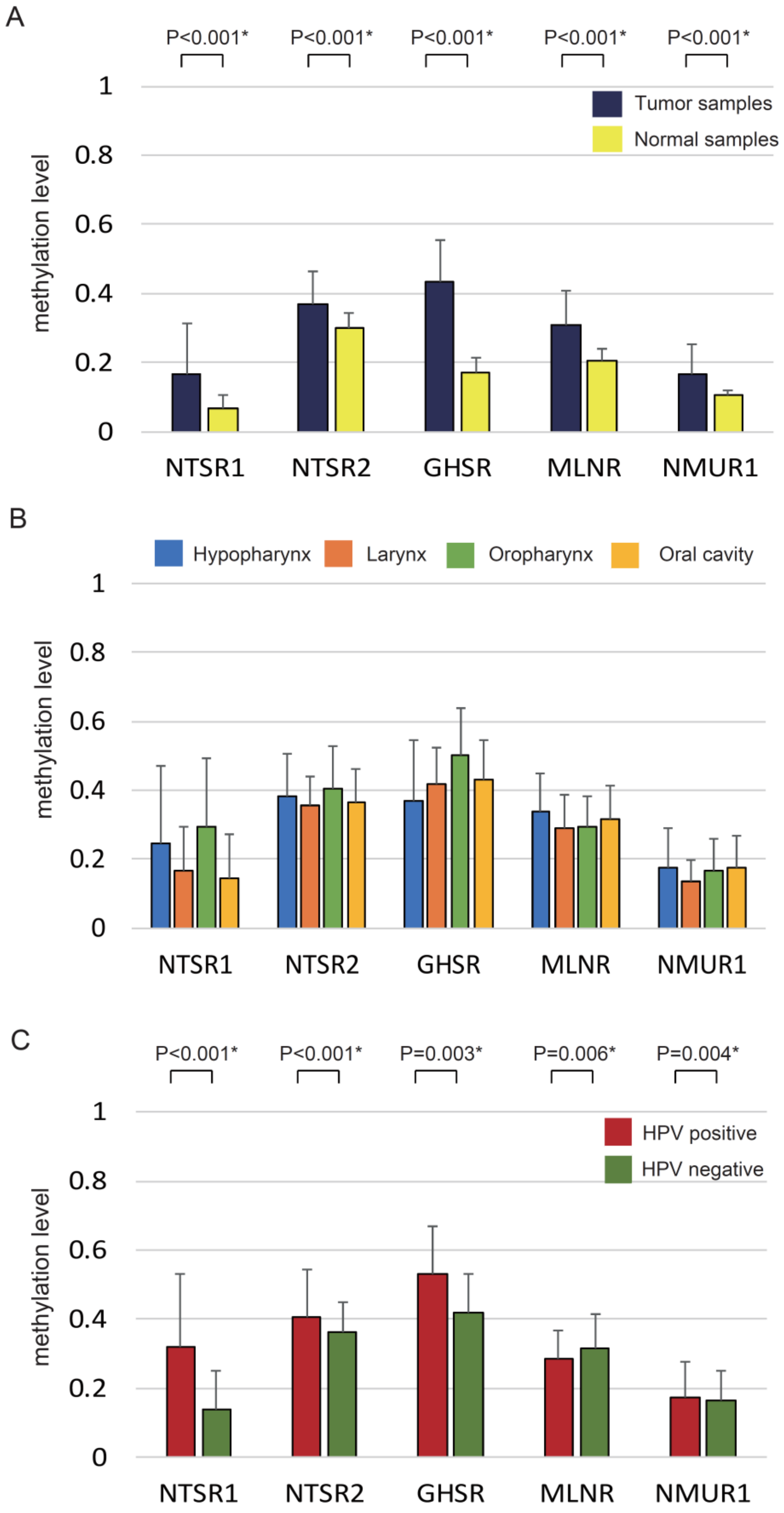
A
P<0.001*
P<0.001*
P<0.001*
P<0.001*
P<0.001*
Tumor samples
Normal samples
methylation level
1
0.8
0.6
0.4
0.2
0
NTSR1
NTSR2
GHSR
MLNR
NMUR1
B
Hypopharynx
Larynx
Oropharynx
Oral cavity
methylation level
NTSR1
NTSR2
GHSR
MLNR
NMUR1
C
P<0.001*
P<0.001*
P=0.003*
P=0.006*
P=0.004*
HPV positive
HPV negative
methylation level
NTSR1
NTSR2
GHSR
MLNR
NMUR1

Additional file 9: Figure S5. Methylation status of the five neuropeptide receptor genes in HNSCC and normal samples in the TCGA database

(a) The methylation data for *NTSR1*, *NTSR2*, *GHSR, MLNR,* and *NMUR1* in HNSCC and normal samples were collected from TCGA database. *P < 0.001. (b) Site-Specific methylation patterns of the TCGA data. (c) The methylation data for *NTSR1*, *NTSR2*, *GHSR, MLNR,* and *NMUR1* in HPV-positive HNSCC and HPV-negative HNSCC were collected from the TCGA database. *P < 0.001.

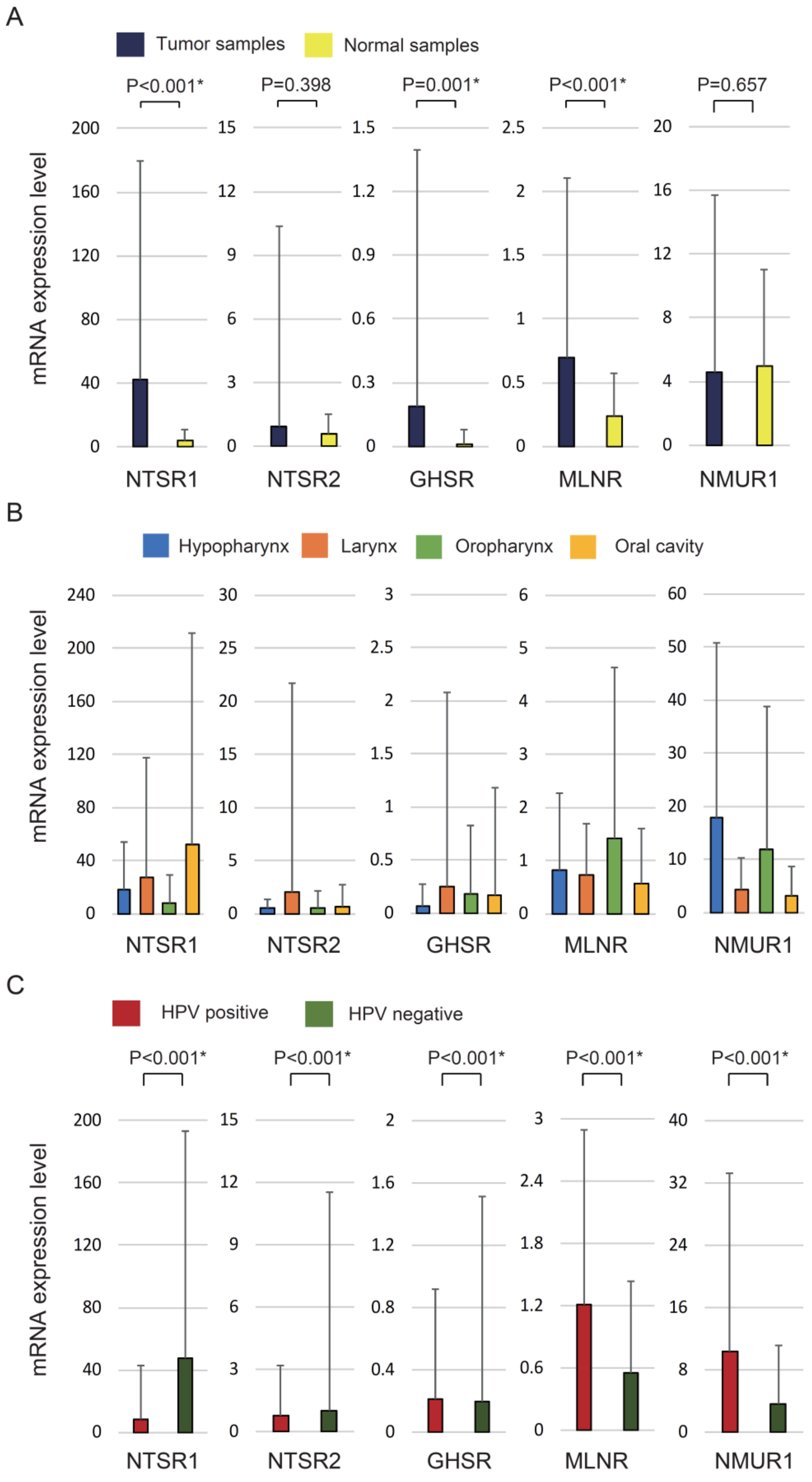
A
Tumor samples
Normal samples
P<0.001*
P=0.398
P=0.001*
P<0.001*
P=0.657
mRNA expression level
NTSR1
NTSR2
GHSR
MLNR
NMUR1
B
Hypopharynx
Larynx
Oropharynx
Oral cavity
mRNA expression level
NTSR1
NTSR2
GHSR
MLNR
NMUR1
C
HPV positive
HPV negative
P<0.001*
P<0.001*
P<0.001*
P<0.001*
P<0.001*
mRNA expression level
NTSR1
NTSR2
GHSR
MLNR
NMUR1

Additional file 10: Figure S6. mRNA expression status of the five neuropeptide receptor genes in HNSCC and normal samples in the TCGA database.

Additional file 11: Table S5. GHSR methylation status, 3q gain, and HPV status in HNSCC from the TCGA database.

Patient and Variable	Cases	GHSR methylation levels ± SD	P-value †
3q gain	218	0.467 ± 0.125	
3q not gain	153	0.410 ± 0.110	0.044*
3q gain and HPV positive	46	0.558 ± 0.123	
3q gain and HPV negative	162	0.443 ± 0.114	0.245

† Student t test * P<0.05
